# Supplementary material for: Estimating Traffic Disruption Patterns with Volunteered Geographic Information
Source: Sci Rep. 2020 Jan 27;10:1271. doi: 10.1038/s41598-020-57882-2 (PMC6985234; doi:10.1038/s41598-020-57882-2)
Supplement: Supplementary file 1 — Supplementary Information. [file 41598_2020_57882_MOESM1_ESM.pdf]

Estimating Traffic Disruption Patterns with Volunteered Geographic Information

Chico Q. Camargo[1], Jonathan Bright[1], Graham McNeill[1], Sridhar Raman[2], Scott A. Hale[1,3,*]

[1]Oxford Internet Institute, University of Oxford, Oxford, United Kingdom
[2]Oxford Brookes University, Oxford, United Kingdom
[3]Alan Turing Institute, London, United Kingdom

[*]scott.hale@oii.ox.ac.uk

Supplementary Information

Variable	Estimate
Commercial	0.0227***
Recreational	-0.0303**
Institutional	0.0087
Green space	0.0026
Industrial	-0.0005
Residential	-0.0084
Observations	112
Adjusted R^2	0.26

(a) Meta-categories only

Variable	Estimate
Parking	0.040***
House	0.005*
...	
Pitch	-0.007
Farmyard	-0.008
Pub	-0.013
Observations	112
Adjusted R^2	0.45

(b) Granular model

* $p < 0.05$, ** $p < 0.01$, *** $p < 0.001$

Table S1: Coefficients for the linear regression model for the incoming traffic for every ward. The traditional land-use categories shown in (a) that produce an adjusted $R^2 = 0.26$, while the granular classifications used in (b) increase the adjusted R^2 to 0.45. Only a small subset of the 40 predictor variables are shown for (b). Entries without * have p-values in excess of 0.05.

	ranking	stability
commercial	1.000	1.000
recreational	1.028	0.972
institutional	1.113	0.915
green space	1.506	0.607
residential	2.048	0.458
industrial	2.839	0.209

(a) Meta-categories, total traffic volume

	ranking	stability
clothes	1.000	1.000
bench	1.000	1.000
supermarket	1.022	0.978
post box	1.146	0.876
playground	1.317	0.829
fast food	1.525	0.792

(b) Granular model, total traffic volume

Table S2: Average ranking and stability of different meta-categories in predicting the traffic volume for every Oxfordshire ward, for trips at any time of the day. Only the top 6 OSM categories are shown in (b). The ranking and stability results are similar to the ones obtained when only selecting trips on weekday mornings.

OSM category	Coefficient	OSM category	Coefficient	OSM category	Coefficient	OSM category	Coefficient
allotments	0.0778 **	farmland	0.555 ***	park	-0.011	residential	0.6092 ***
apartments	-0.0908 **	farmyard	0.3094 ***	parking	0.2406 ***	restaurant	0.0079
bench	0.0372	fast food	0.0502	pitch	0.0811 *	retail	0.0482
bicycle parking	0.029	forest	0.236 ***	place of worship	0.0088**	school	0.0422**
cafe	-0.0727 *	garage	-0.0146	playground	-0.028	soccer	0.0937 *
clothes	0.0659	garden	-0.067	post box	0.0786 **	supermarket	-0.0689 *
commercial	0.0765 **	grass	0.0241	pub	-0.0425	telephone	-0.0291
convenience	-0.0028	house	0.0639 *	recreation ground	0.0269	tennis	0.071
entrance	-0.0453	industrial	0.1388 ***	recycling	-0.0028	terrace	-0.0426
farm	0.2515 ***	meadow	0.1846 ***	reservoir	0.1166	university	0.1179 **

Table S3: All coefficients of the granular model of traffic disruptions.
Respectively, *, ** and *** indicate $p < 0.05$, $p < 0.01$ and $p < 0.001$.
